# Molecular detection of *Enterobacter hormaechei* in bovine respiratory disease

**DOI:** 10.17221/54/2024-VETMED

**Published:** 2024-12-27

**Authors:** Hasanain A. J. Gharban

**Affiliations:** Department of Internal and Preventive Veterinary Medicine, College of Veterinary Medicine, University of Wasit, Wasit, Iraq

**Keywords:** antibiotic sensitivity test, *Enterobacter cloacae* complex, Iraq, phenotypic examination, phylogenetic analysis, undifferentiated fever

## Abstract

Bovine respiratory disease (BRD) develops from complex interactions among environmental, host and pathogenic factors. This study aimed to phenotypically identify *Enterobacter hormaechei* isolated from cattle with BRD and assess antimicrobial susceptibility and determining the molecular phylogeny of local *E. hormaechei* strains. Between November 2023 and March 2024, nasal swabs were collected from 93 cattle with BRD, before culturing for phenotypic analysis, and performing the polymerase chain reaction (PCR) for molecular characterisation. Of the 93 samples evaluated, 15.79% and 24.56% tested positive for *E. hormaechei* isolates on culture and PCR, respectively. The local isolates exhibited high resistance to amoxicillin, ampicillin, amikacin, nalidixic acid and ceftazidime; high susceptibility to azithromycin, levofloxacin, gentamicin, ofloxacin, cefepime, ceftriaxone, cefotaxime, nitrofurantoin, ceftazidime and ciprofloxacin; and moderate susceptibility to ciprofloxacin, colistin, imipenem and meropenem. Multiple sequence alignment, phylogenetic tree analysis and homology sequence identification, showed that the five positive isolates were similar to the reference isolate. To the best of our knowledge, this is the first time that *E. hormaechei* has been isolated in cattle with BRD in Iraq. Because phenotype-based assays show limited accuracy to identify species, we recommend molecular and phylogenetic analysis be included in all similar studies in the future.

Complex interactions among host, pathogenic and environmental factors can act as stressors that enhance the transmission of infectious agents between animals and promote the subsequent development of infection (Tshinavhe 2019; Zhou et al. 2023; Gharban and Ajaj 2024). Bovine respiratory disease (BRD), also known as undifferentiated fever, is a respiratory illness with a multifactorial aetiology, specifically, a viral infection that can compromise the immune system in the affected cattle, leading to bacterial infection of the lower respiratory system (Alexander et al. 2020; Swoboda 2023). The levels of morbidity and mortality associated with BRD highly depend on an array of risk factors, including transport, crowding, dust, inadequate ventilation, inclement weather and commingling (da Silva 2017; Kamel et al. 2024). The use of various antibiotics is the first step in the treatment of respiratory infections, although some cases may not respond because of failure to accurately diagnose BRD (Coetzee et al. 2019; Coetzee et al. 2020; Cummings et al. 2022). *Enterobacter hormaechei* is a non–spore-forming, motile, facultative, anaerobic, Gram-negative bacterium belonging to the family *Enterobacteriaceae* within the phylum *Pseudomonadota* (Wang et al. 2020a; Wang et al. 2020b; Wu et al. 2020). This bacterium was first isolated in humans by O’Hara et al. (1989) from the sputum of a male patient. Qiu et al. (2024) later proposed that it should be a member of the Enterobacter cloacae complex (ECC). Like other *Enterobacter* spp., *E. hormaechei* has been isolated from the intestines of humans and animals and various environmental niches, including plants, water and soil (Cao et al. 2022). Recently, *E. hormaechei* was implicated in several diseases and predominated multidrug-resistant ECC species (Yeh et al. 2022).

The phenotypic assays routinely used in most laboratories have failed to detect various ECC species because of a lack of standardised methods and consensus in identifying *E. hormaechei* (Yeh et al. 2022; Doern 2024). Molecular methods, notably the polymerase chain reaction (PCR), and the use of marker genes, such as the *16S rRNA* gene, have made the characterisation of *E. hormaechei* in various environmental and clinical samples easier and more reliable (Sutton et al. 2018; Zhang et al. 2021; Lipworth et al. 2022). Additionally, the use of phylogenetic analysis has increased the number of reports of emerging antibiotic-resistant *E. hormaechei* strains (Cardoso et al. 2022; Leighton et al. 2024).

In Iraq, *E. hormaechei* has previously been detected in clinical samples, environmental samples, hospital food samples (Abbas and Radhi 2016), frozen meat (Husain and Aziz 2022), human cases of diarrhoea (Al-Saadi and Abbas 2020) and human cases of gingivitis (Khalaf et al. 2023). Therefore, this study aimed to phenotype *E. hormaechei* strains isolated from cattle with BRD and determine the antimicrobial susceptibility and molecular phylogeny of local *E. hormaechei* strains.

## MATERIAL AND METHODS

### Clinical examination and sample collection

Between November 2023 and January 2024, nasal swab samples were obtained from 57 cattle that exhibited undifferentiated respiratory signs, including pneumonia; watery, mucoid, or mucopurulent nasal discharge; dyspnoea; elevated respiratory rate; shallow breathing; coughing-with or without other clinical signs, such as fever, depression, loss of appetite, restlessness, lacrimation and diarrhoea or constipation. The samples were transported to the laboratory in labelled plastic tubes containing 4 ml of Luria-Bertani broth (SRL Chemicals, Mumbai, India), before they were subjected, as soon as possible, to bacterial isolation and molecular analysis.

### Phenotypic assays

In the laboratory, each swab sample was incubated for 5 h at 37 °C, before 100 μl of it was spread on plates with Luria-Bertani agar (SRL Chemicals, Mumbai, India) supplemented with enrofloxacin (1 μg/ml). After overnight incubation at 37 °C, the suspected *Enterobacter* sp. colonies were tested biochemically to identify the species and re-cultured on Mueller-Hinton Agar (SRL Chemicals, Mumbai, India) for antimicrobial susceptibility testing.

### Antimicrobial susceptibility testing

The disc diffusion test, as described by Khan et al. (2019), was used to examine the susceptibility of bacterial cultures to 17 antibiotics of various groups. The test panel of antibiotics, all obtained from Riverside Medical (Ahmedabad, India), included the following: amikacin (30 μg), amoxicillin (30 μg), ampicillin (100 μg), azithromycin (10 μg), cefepime (30 μg), cefotaxime (30 μg), ceftazidime (30 μg), ceftriaxone (30 μg), ciprofloxacin (10 μg), colistin (30 μg), gentamicin (10 μg), imipenem (10 μg), levofloxacin (5 μg), meropenem (10 μg), nalidixic acid (10 μg), nitrofurantoin (10 μg) and ofloxacin (10 μg). The results were interpreted according to the Clinical and Laboratory Standards Institute (CLSI) breakpoints (Hsueh et al. 2010).

### Molecular assay

DNA was extracted from the swab samples according to the Type G Protocol of the G-Spin Total DNA Extraction Kit (iNtRON Biotechnology, Seongnam, Republic of Korea). The Nanodrop System (Thermo Fisher Scientific, Loughborough, UK) was used to determine the concentration and purity of each DNA sample. For amplification, one set of primers, based on the National Center for Biotechnology Information (NCBI) GenBank sequence data for an *E. hormaechei* isolate (ID: AM947046.1), were designed for this study [HAF: (5'-CGG TAG CTA ATA CCG CAT AAC G-3') and HAR: (5'-CTT CCT CCC CGC TGA AAG TA-3')]. For PCR, the Go *Taq* Green Master Mix Kit (Promega, Madison, WI, USA) was used according to the manufacturer’s instructions. Tubes were prepared with Master Mix, with a final volume of 20 μl, and placed in a thermal cycler (T100; Bio-Rad, Hercules, CA, USA). The amplification conditions consisted of an initial denaturation at 95 °C for 7 min, 35 cycles of denaturation at 95 °C for 30 s, annealing at 57 °C for 30 s, extension at 72 °C for 30 s, and a final extension at 72 °C for 10 minutes. The PCR products were analysed by electrophoresis at 100 V and 80 A for 90 min on an agarose gel stained with ethidium bromide. The positive PCR products were visualised with a UV transilluminator (WiseD; Daihan Scientific Co., Wonju, Republic of Korea) at 291 bp and photographed with a digital camera.

### Phylogeny

Five positive DNA samples were sent to a private company (Macrogen, Seoul, Republic of Korea) for sequencing. Sequences were analysed with MEGA7 software v11 (http://www.megasoftware.net) to perform the phylogenic tree analysis and homology sequence identification, comparing the local and reference (NCBI GenBank) *E. hormaechei* isolates.

### Statistical analysis

To analyse the data obtained in this study, a one-way analysis of variance was used for comparison parameters among three or more treatment groups and the *t*-test was used for comparisons between two groups. Statistical analysis was performed with GraphPad Prism software v9.0.1 (GraphPad Software Inc., San Diego, CA, USA). Values of *P* < 0.05 were considered statistically significant.

## RESULTS

**Figure 1:**
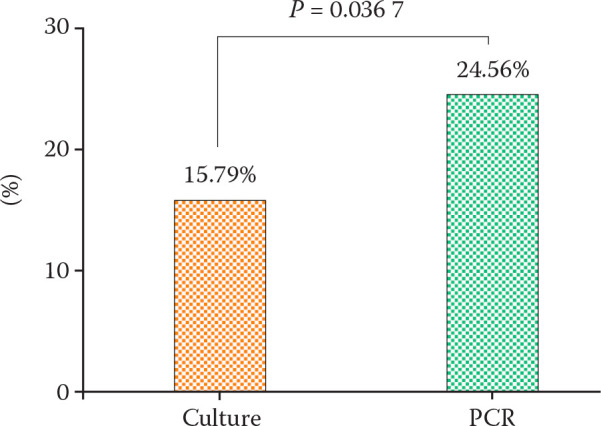
Isolates positive for *Enterobacter hormaechei* on culture and conventional polymerase chain reaction (PCR)

**Figure 2:**
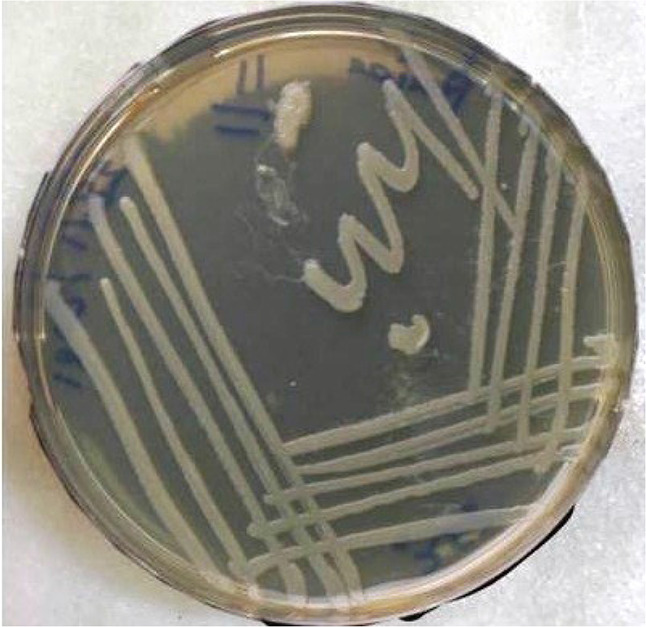
Colonies of *Enterobacter hormaechei* isolates on Luria-Bertani agar

**Figure 3:**
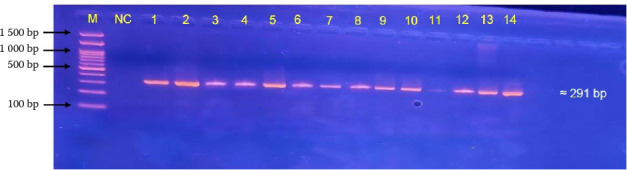
Agarose gel electrophoresis of polymerase chain reaction (PCR) products of the *16S rRNA* gene: ladder marker (M, 100–1 500 bp), negative control (NC), and isolates positive for *E. hormaechei* at approximately 291 bp (lanes 1–14)

Of the 57 cattle diagnosed with BRD, 9 (15.8%) and 14 (24.6%) tested positive for *E. hormaechei* on culture and PCR, respectively (Figures 1–3). Antimicrobial susceptibility testing (Table 1) revealed that all the nine (100%) *E. hormaechei* isolates were resistant to amoxicillin plus ampicillin; 55.56% were resistant to amikacin plus nalidixic acid; and 44.44% were resistant to ceftazidime (*P* < 0.007 for all). Of these same isolates, 55.56% were susceptible to ciprofloxacin (55.56%) and 44.44% were susceptible to the combination of colistin, imipenem and meropenem (*P* < 0.0001 for all). However, higher susceptibility was observed to some combinations of antibiotics: azithromycin plus levofloxacin (89.89%), gentamicin plus ofloxacin (77.78%), cefepime plus ceftriaxone (66.67%), cefotaxime plus nitrofurantoin (55.56%) and ceftazidime plus ciprofloxacin (44.44%). The differences in percentage susceptibilities among antibiotics were significant (*P* < 0.000 1 for all).

**Table 1:** Testing for antimicrobial susceptibility of nine isolates that were positive for *Enterobacter* sp. on culture

Antibiotic	Zone of inhibition (mm)			Pattern n (%)			P-value
	resistant	susceptible		resistant	intermediate	susceptible	
Amikacin	≤ 14	≥ 17		5 (55.6)*	2 (22.2)	2 (22.2)	0.040 1
Amoxicillin	≤ 15	≥ 20		9 (100)****	0 (0)	0 (0)	0.000 1
Ampicillin	≤ 10	≥ 14		9 (100)****	0 (0)	0 (0)	0.000 1
Azithromycin	≤ 12	≥ 15		0 (0)	1 (11.1)	8 (89.9)***	0.000 2
Cefepime	≤ 17	≥ 25		1 (11.1)	2 (22.2)	6 (66.7)**	0.004 1
Cefotaxime	≤ 17	≥ 22		3 (33.3)	1 (11.1)	5 (55.6)**	0.009 8
Ceftazidime	≤ 20	≥ 21		4 (44.4)**	1 (11.1)	4 (44.4)**	0.009 1
Ceftriaxone	≤ 14	≥ 20		0 (0)	3 (33.3)	6 (66.7)**	0.008 7
Ciprofloxacin	≤ 21	≥ 26		0 (0)	5 (55.6)**	4 (44.4)	0.009 1
Colistin	≤ 10	≥ 11		3 (33.3)	4 (44.4)*	2 (22.2)	0.023 8
Gentamicin	≤ 11	≥ 15		0 (0)	2 (22.2)	7 (77.8)**	0.008 1
Imipenem	≤ 19	≥ 23		3 (33.3)	4 (44.4)*	2 (22.2)	0.023 8
Levofloxacin	≤ 16	≥ 21		0 (0)	1 (11.1)	8 (88.9)***	0.000 2
Meropenem	≤ 14	≥ 18		2 (22.2)	4 (44.4)*	3 (33.3)	0.023 8
Nalidixic acid	≤ 13	≥ 19		6 (66.7)**	2 (22.2)	1 (11.1)	0.004 1
Nitrofurantoin	≤ 14	≥ 17		1 (11.1)	3 (33.3)	4 (55.6)**	0.009 8
Ofloxacin	≤ 12	≥ 16		0 (0)	2 (22.2)	7 (77.8)**	0.008 1
							
*P*-value	–	–		0.007	0.000 1	0.000 1	–

Significance: *P < 0.05; **P < 0.01; ***P < 0.001; ****P < 0.000 1

**Figure 4:**
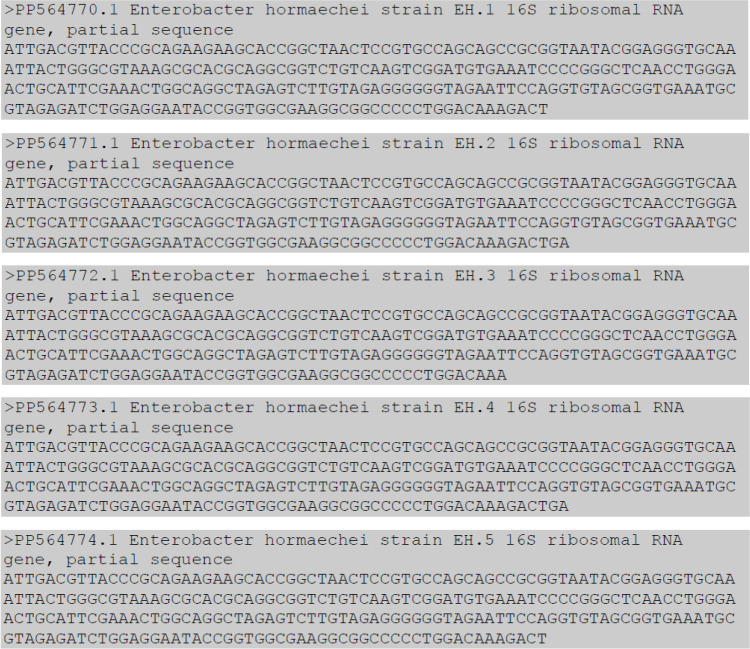
Sequences of local *E. hormaechei* isolates deposited into the National Center for Biotechnology Information GenBank

**Figure 5:**
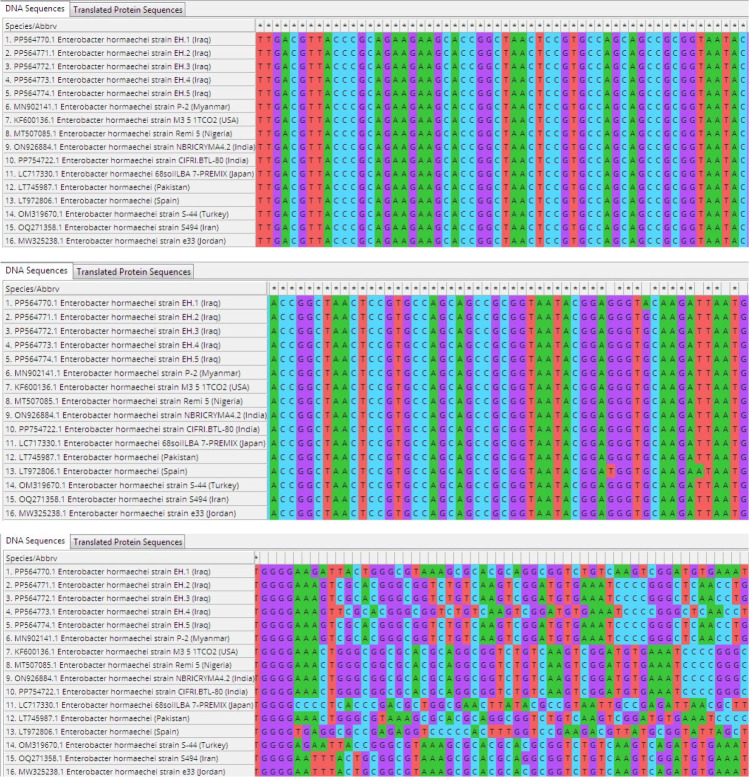
Multiple se-quence alignment of local and reference (National Center for Biotechnology Information GenBank) *E. hormaechei* isolates

**Figure 6:**
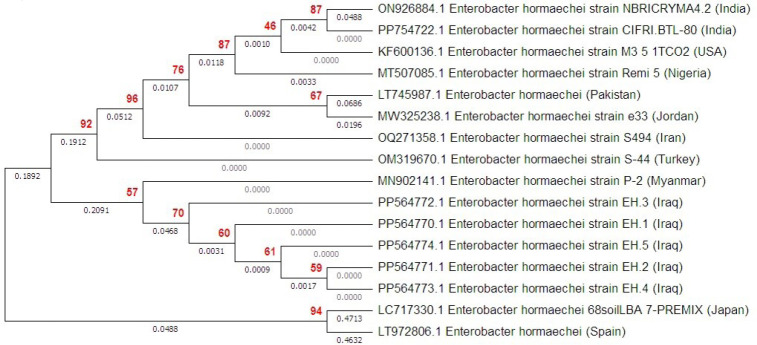
Phylogenetic tree of local and reference (National Center for Biotechnology Information GenBank) *E. hormaechei* isolates

The DNA of five *E. hormaechei* isolates was sequenced and the sequences were deposited in the the NCBI GenBank under the following accession numbers (Figure 4): PP564770.1, PP564771.1, PP564772.1, PP564773.1 and PP564774.1. Multiple sequence alignment, phylogenetic tree analysis and homology sequence identification revealed that the local *E. hormaechei* isolates, relative to the reference NCBI Basic Local Alignment Search Tool isolate (MN902141.1, from Myanmar) showed a proportion of mutations/changes ranging from 0.1% to 0.6% and similarity ranging from 96.23% to 96.84% (Figures 5 and 6; Table 2).

**Table 2:** Homology sequence identification for local and National Center for Biotechnology Information Basic Local Alignment Search Tool *Enterobacter hormaechei* isolates

Local isolates		NCBI-BLAST isolate			Identity (%)
Name	accession no.	species	country	accession no.	
EH.1	PP564770.1	*E. hormaechei*	Myanmar	MN902141.1	96.84
EH.2	PP564771.1				96.84
EH.3	PP564772.1				96.23
EH.4	PP564773.1				96.23
EH.5	PP564774.1				96.84

## DISCUSSION

Cattle are susceptible to several respiratory diseases, with various aetiologies (Constable et al. 2016). One of the most common respiratory diseases is BRD, which severely affects livestock because it damages the respiratory tract, causes systemic illness, reduces daily weight gain, lowers the feed conversion rate and can kill (Lima et al. 2016; Theurer et al. 2021). Although many studies have detected *E. hormaechei* in faeces, urine, sputum, skin, bile and blood samples (Davin-Regli et al. 2019; Munson and Carroll 2019; Moxley 2022), it is still an uncommon causative agent of respiratory infections. In this study, phenotypic and molecular analyses showed positivity for *E. hormaechei* in about 16% and 25% of the animals with BRD, respectively. Many previous studies have focused on the isolation of *E. hormaechei* from clinical samples (Wenger et al. 1997; Gravey et al. 2020; Martins et al. 2020). In humans, *E. hormaechei* is of clinical importance in immunocompromised patients and has been linked to outbreaks of sepsis in neonatal intensive care units (Martins et al. 2020; Ramirez and Giron 2020; Cao et al. 2022). In animals, *E. hormaechei* has been detected in faeces of piglets (Shi et al. 2022); in cases of uterine infection in foxes (Wen et al. 2017); in septic arthritis in a green sea turtle (Goldberg et al. 2019); from lung and nasal secretion samples collected from calves (Wang et al. 2020b); in urinary tract infection in a cat (Hayakawa Ito de Sousa et al. 2021); in cases of respiratory disease complex in pets (Khalifa et al. 2020); from a vaginal swab collected from a cow (Zaitsev et al. 2022); in chickens (Amusan 2023); and in the rumen of a dairy cow (Zhong et al. 2023).

Significant differences between traditional and molecular methods, in terms of positivity rates, have been reported. These differences could be attributed to the fact that traditional (phenotypic) methods are time-consuming and depend on growth, which limits their clinical utility, and that their results are subjective, whereas molecular assays are performed directly and rapidly with high sensitivity and specificity, making them more accurate in the detection of resistance genes and allowing earlier administration of targeted therapy (AlTamimi et al. 2017; Gajic et al. 2022).

Antibiotic resistance, regulation of resistance genes and clinical implications have been extensively studied in *Enterobacter* spp. (Davin-Regli et al. 2019; Gravey et al. 2020; Chang et al. 2022; Leelapsawas et al. 2024; Sabtcheva et al. 2024). Our findings show that the local *E. hormaechei* isolates were highly resistant to amoxicillin plus ampicillin, amikacin, nalidixic acid plus ceftazidime, whereas they were highly susceptible to azithromycin, levofloxacin, gentamicin, ofloxacin, cefepime, ceftriaxone, cefotaxime, nitrofurantoin, ceftazidime and ciprofloxacin, moderately susceptibility to ciprofloxacin, colistin, imipenem and meropenem. The virulence of *E. hormaechei* might be higher than that of other ECC species because of many pathogenicity islands that occur on its chromosomes (Mavroidi et al. 2023). The first strain of *E. hormaechei*, isolated by O’Hara et al. (1989), was susceptible or moderately susceptible to amikacin, azlocillin, cefotaxime, ceftazidime, ceftriaxone, chloramphenicol, gentamicin, mezlocillin, moxalactam, piperacillin, trimethoprim-sulfamethoxazole, sulfisoxazole, thienamycin, tobramycin and trimethoprim. Al-Saadi and Abbas (2020) recorded an *E. hormaechei* strain that was resistant to amikacin, ampicillin, amoxicillin/clavulanate, cefixime, cefotaxime, cefoxitin, ceftazidime, cephalothin, chloramphenicol, ciprofloxacin, erythromycin, imipenem, streptomycin and ticarcillin/clavulanate. Zaitsev et al. (2022) reported *E. hormaechei* strains showing susceptibility to at least two beta-lactam antibiotics (cefquinome and meropenem), but being resistant to eight other groups of antibiotics. Yeh et al. (2022) observed an increasing prevalence of clinical isolates of *E. hormaechei* with varying degrees of resistance to amikacin, aztreonam, carbapenems, cefotaxime, cefoxitin, ceftazidime, ciprofloxacin, colistin/gentamicin, piperacillin/tazobactam, tigecycline, tobramycin and trimethoprim/sulfamethoxazole. The emergence of *E. hormaechei* isolates that are resistant to several antibiotics might be attributed to the presence of various resistance genes and the production of antibiotic lysis enzymes, such as carbapenemases and β-lactamases.

Based on the *16S rRNA* gene, phylogenetic analysis of study *E. hormaechei* isolates revealed their identity to be comparable to that of the Myanmar *E. hormaechei* isolate. Widespread use of marker genes, particularly the *16S rRNA* gene, has allowed new bacterial species and subspecies to be identified (Sutton et al. 2018). Although *E. hormaechei* has been identified as the predominant species of the genus in clinical *Enterobacter* isolates (Hoffmann and Roggenkamp 2003; Hoffmann et al. 2005; Paauw et al. 2009; Ohad et al. 2014; Guerin et al. 2016), many GenBank submissions, clinicians, and articles have misidentified these strains as *E. cloacae*. This might be due to the fact that *E. hormaechei* subspecies had not been described in validated studies until recently, perhaps as shorthand for the ECC (Sutton et al. 2018).

In conclusion, this study reports, for first time, isolation of *E. hormaechei* from cattle with BRD and that such isolates have been detected in Iraq. Further studies are needed to investigate the role played by *E. hormaechei* in various infections in cattle, and in other domestic animals and wild animals. As phenotype-based assays show limited accuracy in detecting species, we recommend that molecular testing and phylogenetic analysis be part of similar studies in the future.
